# Ethnoveterinary Survey Conducted in Baiku Yao Communities in Southwest China

**DOI:** 10.3389/fvets.2021.813737

**Published:** 2022-01-25

**Authors:** Binsheng Luo, Qimin Hu, Kedao Lai, Arvind Bhatt, Renchuan Hu

**Affiliations:** ^1^Lushan Botanical Garden, Jiangxi Province and Chinese Academy of Sciences, Lushan, China; ^2^Guangxi Key Laboratory of Traditional Chinese Medicine Quality Standards, Guangxi Institute of Traditional Medical and Pharmaceutical Sciences, Nanning, China

**Keywords:** ethnoveterinary medicine, traditional knowledge, animal plague, ethnobotany, Yao nationality

## Abstract

Baiku Yao is a branch of the Yao ethnic group mainly living in Guangxi and Guizhou provinces of China. They are recognized by UNESCO as an ethnic group with an intact ethnic culture. The Baiku Yao people have extensive ethnoveterinary knowledge, which they used to prevent and control various animal diseases. During the African swine fever outbreak, the livestock of the Baiku Yao community remained unaffected. We investigated ethnoveterinary knowledge among local Baiku Yao villagers. A total of 39 ethnoveterinary plant species are utilized for the treatment of various diseases. Five species, namely, *Stephania kwangsiensis, Aristolochia kwangsiensis, Clerodendrum bungei, Paederia foetida*, and *Tetradium ruticarpum*, had the highest relative frequency values. *Strobilanthes cusia, Tetradium ruticarpum*, and *Stephania kwangsiensis* are highly valued locally for treating animal plagues. The existing traditional ethnoveterinary knowledge needs to be conserved and validated scientifically.

## Introduction

Animal husbandry has played an extremely important role throughout human history by transforming transportation and the economy. China has experienced substantial increases in the demand for and production of livestock products (1). However, the livestock sector is continuously facing various new challenges due to emerging new diseases, which have direct impacts on livestock producers. Recently, animal disease outbreaks and epidemics have increased in frequency and caused substantial losses to the animal husbandry industry (2). Consequently, the use of veterinary drugs to protect and maintain animal health is also increasing. However, this poses a severe risk to human health due to various side effects (3, 4). Chemical compounds in veterinary drugs may persist in livestock meat, thus increasing the possibility of human exposure through meat consumption, and some compounds can become enriched in the human body (5), resulting in health risks. Similarly, the abuse of antibiotics can also lead to the transmission of pathogenic bacteria to humans through direct animal contact or food consumption (6). Moreover, both pathogenic and non-pathogenic bacteria carrying antimicrobial resistance genes can contaminate the environment through livestock excrement (7), which ultimately accelerates zoonoses (8).

To solve these problems, some countries have already deployed corresponding countermeasures. For example, the Chinese government has banned the application of 11 growth-promoting antibiotics in animal feed since 2020 (9). Utilizing ethnoveterinary medicines could be a beneficial alternative; thus, the study of traditional ethnoveterinary drugs is receiving tremendous attention (10). Ethnoveterinary medicine is the general scientific term for traditional animal health care and is a cheaper, easier, and more sustainable alternative to synthetic drugs and medicines. Related traditional community-based epidemic prevention concepts, such as physical isolation and natural feed selection, also have certain scientific connotations. However, traditional ethnoveterinary knowledge is at risk of extinction due to modern technology and lifestyle changes (11). Therefore, there is an urgent need to scientifically record and verify ethnoveterinary medical knowledge (12).

During former field trips to some Baiku Yao villages and formal discussions with local people, we found that that their livestock was relatively unaffected by the African swine fever outbreak in 2019, even though most local people did not have access to modern veterinary medicine. In mainland China, African swine fever reduced pig production by up to 40% (13). This led us to investigate ethnoveterinary knowledge among the local Baiku Yao villagers. We hypothesized that Baiku Yao villagers might have unique knowledge about traditional veterinary medicines that helped to control the epidemic and ensure livestock safety. Therefore, in this study, we focused on documenting traditional ethnoveterinary knowledge and practices.

Baiku Yao is a branch of the Yao ethnic group, with more than 30,000 people mainly living in Guangxi and Guizhou provinces of China. They are known for their unique clothing, and white trousers are the most unique feature of men's clothing (14, 15). The Baiku Yao have been nicknamed the “living fossil of human civilization,” and United Nations Educational, Scientific and Cultural Organization (UNESCO) recognizes them as an ethnic group with a mostly intact ethnic culture (15). Animal husbandry and crop planting play important roles in the Baiku Yao culture and their livelihoods. Usually, each family raises livestock, including cattle and pigs, for their meat, hides, wool, and milk, and as work animals. However, an increase in agricultural mechanization and transportation infrastructure has markedly reduced the need for animal labor; therefore, the Baiku Yao people are now using livestock mostly as food and economic resources. The local government is encouraging and financially supporting the Baiku Yao people in raising livestock for poverty alleviation.

Accordingly, this study aimed to verify our hypothesis that Baiku Yao villagers have unique epidemic prevention knowledge and traditional veterinary medicine resources to ensure livestock safety. Additionally, we hope to present this classic case to promote new related scientific research and development.

## Methods

### Study Sites

**Figure 1:**
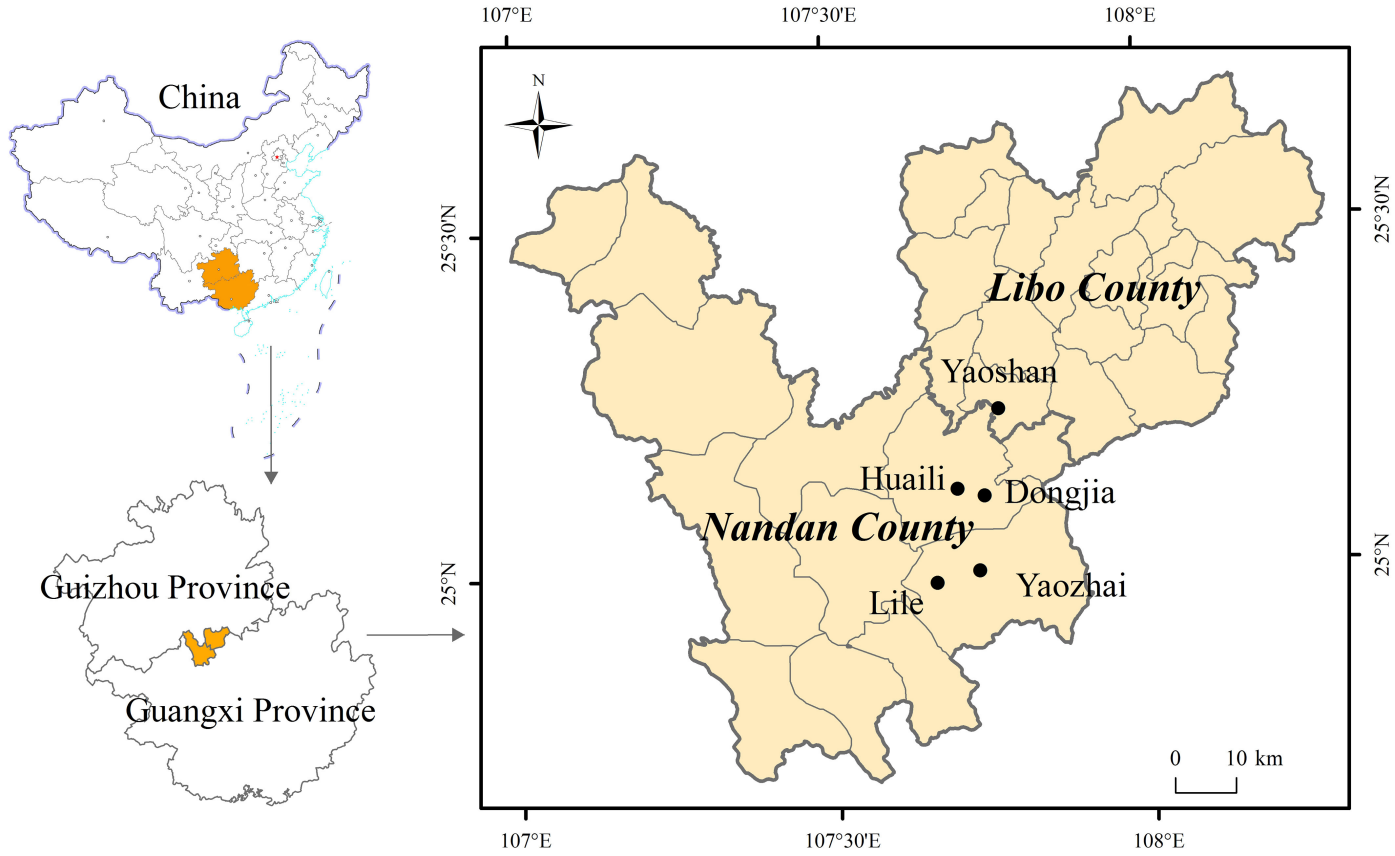
The study area.

The Baiku Yao population is mainly distributed in Lihu and Baxu townships in Nandan County (Guangxi Province) and Yaoshan township in Libo County (Guizhou Province), China (15). However, some Baiku Yao people are scattered in the Celing township of Jinchengjiang District and Mangchang town of Nandan County in Guangxi, China (15). The southeast corner of the Yunnan-Guizhou Plateau, where the Baiku Yao people are located, has a subtropical monsoon climate, with rich plant biodiversity. We selected a total of five villages, namely, Yaozhai and Lile village (Baxu township), Huaili, and Dongjia village (Lihu township), and Yaoshan village (Yaoshan township), to explore traditional ethnoveterinary knowledge (Figure 1). These villages were selected based on the distribution of Baiku Yao villages and the recommendation of local officials from Nandan County and Libo County.

### Data Collection

Ethnobotanical data were collected using different methods, such as key informant interviews, casual interviews, informal discussions and semistructured interviews (16). Before the interview, every participant was informed about the nature and purpose of the research. We selected a total of 53 inhabitants for detailed interviews (27 females and 26 males), including Baiku Yao community cadres, community doctors, traditional folk herbalists, professional livestock breeders, and traditional farmers whose livestock remained unaffected by the African swine fever outbreak of 2019. Of the 53 people, 23 key informants were recommended by local government officials, Baiku Yao National Museum staff and village leaders. Thirty informants were chosen using the snowball sampling method at the local market or Baiku Yao village. The informants' ages ranged from 17 to 85 years. We also recorded the basic details of the interviewees, such as age, gender, occupation, etc. The statistics of the participants in each age group are shown in Table 1.

**Table 1:** Demographic information of the participants.

**Age group**	**Male**	**Female**	**No. of people (percentage)**
≥70	3	6	9 (16.98)
60–69	5	8	13 (24.53)
50–59	6	7	13 (24.53)
40–49	7	4	11 (20.75)
<40	5	2	7 (13.21)
Total	26	27	53 (100)

The taxonomic information, vernacular name, useful plant part, usage and distribution of the veterinary medicinal plants were documented during the interview. The questions we asked mainly included: (1) What plants do you use to treat animal diseases? (2) What is the local name/medicinal part/medicinal function of this plant? and (3) How do you use this plant (processing methods)?

We also collected herbarium voucher specimens or took voucher photos during the field walks with the participants. The authors later identified the voucher specimens based on consultations with local people or identification using the *Flora of China* (http://www.efloras.org/) and other online databases (http://www.iplant.cn/frps, http://www.cfh.ac.cn/, https://www.gbif.org/, http://www.nsii.org.cn/2017/home.php, http://www.theplantlist.org/, http://ppbc.iplant.cn/). Both the *Flora of China* and the *Angiosperm Phylogeny Group IV system* (APG IV system) were used to determine the scientific name of the plants.

### Data Analysis

The relative frequency of citation (RFC) was used to evaluate the importance of each species (17). The equation to calculate the RFC is as follows: RFC = FC/N, where FC is the number of informants who cited the use of a particular species, and N is the total number of informants (17).

Grading and ranking (GR) were used to assign scores to the plants used for preventing or treating animal diseases, such as African swine fever. Finally, the potential value of each plant species was evaluated according to the total score obtained (18, 19). The principles of scoring were as follows:

(1) One point was assigned for each village that mentioned the use of the target plant species for treating animal disease;
(2) One point was assigned if within the same village, only 2–3 participants mentioned the target plant species for the treatment of animal disease;
(3) Two points were assigned if within the same village, 4 or more participants mentioned the target plant species for the treatment of animal disease [If meet the condition of (3), no point should be added from (2) repetitively].
(4) Two points were assigned if there were related documents mentioning the target plant species or verifying its function in the treatment of animal disease;
(5) Two points were assigned if the identified species had recorded similar usage in other regions. For this, we consulted the available literature.

The higher scoring species represent greater potential research value. Conversely, the lower scoring species have lower potential value. Finally, the ethnoveterinary species for treating animal plague are identified based on grading and ranking methods.

## Results and Discussion

### Taxonomic Distribution

In total, 39 ethnoveterinary plant species belonging to 27 families and 38 genera were reported (Table 2). Out of the 27 plant families, 15 families included only 1 species (accounting for 70.37%). Seven families each contained 2–4 species (accounting for 25.93%), and 1 family (Asteraceae) contained more than 5 species (accounting for 3.7%). The Asteraceae had the largest number of genera (1,600) and species (23,000) in the world (20). Species from the Asteraceae have been indicated to have the most prominent bioactivity, such as anti-inflammatory, antioxidant, and antibacterial activities, and thus have potential applications for use in the pharmaceutical industry (20). However, at the genus level, only 1–2 species per genus were noted (Table 2). These findings indicate that ethnoveterinary plant species are not concentrated in certain plant families or genera, reflecting that the Baiku Yao people have very deep traditional knowledge about animal health care and a comprehensive understanding of surrounding plants.

**Table 2:** Ethnobotanical inventory of the ethnoveterinary plants used by the Baiku Yao people.

**Voucher ID**	**Scientific name**	**RFC**	**Family name**	**Venacular name**	**Application**	**Used parts**	**Use method**	**Collection type**	**GR score**
HRC791	*Curculigo capitulata* (Lour.) Kuntze	0.40	Hypoxidaceae	zu lie li	Swine, cattle: parasites	Whole plant	Mixed with feed	Wild	
HRC160	*Stephania kwangsiensis* H. S. Lo	0.38	Menispermaceae		Swine, cattle: indigestion and plague	Tuber-roots	Decoction and oral	Wild or cultivated	9
HRC153	*Aristolochia kwangsiensis* Chun et F. C. How ex C. F. Liang	0.32	Aristolochiaceae		Swine, cattle: indigestion and diarrhea	Tuber	Decoction and oral	Wild or cultivated	6
HRC163	*Clerodendrum bungei* Steud.	0.26	Lamiaceae	zhu sha	Swine, cattle, chicken: plague	Leaf	External	Wild	6
HRC492	*Paederia foetida* L.	0.25	Rubiaceae	ge ba	Cattle: diarrhea; dog, cattle, swine: plague	Whole plant	Plague: hang on dogs' neck or let the animal sleep on it; indigestion: decoction for oral	Wild	4
HRC568	*Tetradium ruticarpum* (A. Juss.) Hartley	0.23	Rutaceae	mi la	Swine, cattle: plague, indigestion	Branches and fruits	Indigestion: decoction and oral; plague: let animal sleep on it.	Wild or cultivated	9
HRC799	*Strobilanthes cusia* (Nees) Kuntze	0.23	Acanthaceae	yin zhei	Swine: external wounds; swine, cattle: plague	Leaf	External (mixed with Gynura japonica, wine, salt, mud)	Wild	10
HRC810	*Eupatorium fortunei* Turcz.	0.23	Asteraceae	zu niong biao	Swine, cattle: internal injury, plague	Leaf	Mashed for oral	Cultivated	7
HRC560	*Acorus calamus* L.	0.17	Araceae		Chicken, cattle: plague	Whole plant	Decoction and oral	Cultivated	6
HRC783	*Rumex nepalensis* Spreng.	0.17	Polygonaceae		Cattle: bone fracture	Leaf	External	Wild	
HRC493	*Melastoma malabathricum* L.	0.15	Melastomataceae		Cattle: diarrhea	Root	Decoction and oral	Wild	
HRC545	*Glochidion puberum* (L.) Hutch.	0.15	Phyllanthaceae	bai jie	Swine, cattle: diarrhea	Root	Decoction and oral	Wild	
HRC784	*Begonia grandis* Dry.	0.15	Begoniaceae	zhun ca	Cattle: cough, pneumonia	Whole plant	Decoction and oral	Wild	
HRC792	*Polygonum nepalense* Meissn.	0.15	Polygonaceae	wo niao re	Swine: indigestion	Whole plant	Mixed with feed	Wild	
HRC818	*Gnaphalium japonicum* Thunb.	0.15	Asteraceae		Swine: plague, diarrhea	Whole plant	Mixed with feed	Wild	3
HRC533	*Achyranthes longifolia* (Makino) Makino	0.11	Amaranthaceae	ya gei	Swine, cattle: plague	Tuber-roots	Decocted with rice for oral	Wild	
HRC808	*Acorus macrospadiceus* F. N. Wei et Y. K. Li	0.11	Araceae	xi hang	Swine, cattle: plague	Whole plant	Mixed with feed	Cultivated	4
HRC853	*Nandina domestica* Thunb.	0.11	Berberidaceae	nong ce	Swine, cattle: diarrhea	Whole plant	Decoction and oral	Wild	
HRC495	*Rosa laevigata* Michx.	0.09	Rosaceae	ye bu gei ka	Cattle: diarrhea	Root	Decoction and oral	Wild	
HRC797	*Atropa belladonna* L.	0.09	Solanaceae		Chicken: plague	Fruits and leaves	Mixed with feed	Wild	2
HRC801	*Aconitum carmichaelii* Debeaux	0.09	Ranunculaceae	zuo fi ne	Swine, cattle, chicken: parasites	Tuber	Mixed with feed	Wild	
HRC864	*Osbeckia opipara* C. Y. Wu et C. Chen	0.09	Melastomataceae	wo dao mu	Swine, cattle: diarrhea	Root	Decoction and oral	Wild	
HRC32	*Carpesium abrotanoides* L.	0.08	Asteraceae	yan jie	Cattle: skin infections	Leaf	External	Wild	
HRC532	*Zanthoxylum armatum* DC. var. *armatum*	0.08	Rutaceae		Swine, cattle: plague	Branches and leaves	Decocted with rice for oral	Wild	3
HRC809	*Gladiolus gandavensis* Van Houtte	0.08	Iridaceae		Swine, cattle: plague	Tuber	Mixed with feed	Cultivated	3
HRC233	*Lygodium japonicum* (Thunb.) Sw.	0.06	Lygodiaceae	guo de gei	Swine: skin infections	Whole plant	Medicinal bath	Wild	
HRC573	*Liquidambar formosana* Hance	0.06	Hamamelidaceae	yin mei	Cattle: diarrhea	Leaf	Mashed and soaked in water for oral	Wild	
HRC798	*Citrus limonia* Osbeck	0.06	Rutaceae		Chicken: plague	Leaf	Mixed with feed	Wild	1
HRC812	*Bidens alba* (L.) DC.	0.06	Asteraceae	wo zong	Swine, cattle: plague	Branches and leaves	Mixed with feed	Wild	2
HRC819	*Stachyurus chinensis* Franch.	0.06	Stachyuraceae	gai wa	Swine: plague	Leaf	Decoction and mix with the feed; let animal sleep on it.	Wild	2
HRC833	*Perilla frutescens* (L.) Britton var. *frutescens*	0.06	Lamiaceae	wo bai mi	Swine: diarrhea	Leaf	Chopped freshly to feed animal	Cultivated	
HRC863	*Toricellia angulata* Oliv.	0.06	Toricelliaceae	nong ho jie	Swine, cattle: external wounds	Leaf	External	Cultivated	
HRC494	*Rubus niveus* Thunb.	0.04	Rosaceae	wu pao ging	Cattle: diarrhea	Root	Decoction and oral	Wild	
HRC496	*Callicarpa kwangtungensis* Chun	0.04	Verbenaceae	gei yi bu	Cattle: diarrhea	Whole plant	Decoction and oral	Wild	
HRC64	Rubia wallichiana Decne.	0.04	Rubiaceae	si yao cao	Duck: skin infections	Root	External	Wild	
HRC790	*Cunninghamia lanceolata* (Lamb.) Hook.	0.04	Taxodiaceae	nong zan	Cattle: indigestion	Branches and leaves	External	Cultivated	
HRC800	*Gynura japonica* (Thunb.) Juel	0.04	Asteraceae		Swine: external wounds	Leaf	External (mixed with Strobilanthes cusia, wine, salt, mud)	Wild	
HRC905	*Parepigynum funingense* Tsiang et P. T. Li	0.04	Apocynaceae		Swine, cattle: external wounds	Branches and leaves	External	Wild	
HRC527	*Chirita tribracteata* W. T. Wang var. *tribracteata*	0.02	Gesneriaceae		Swine: plague	Whole plant	Cooked as feed	Wild	1

### Life Forms and Used Parts

Most of the plant species identified by the inhabitants as having ethnoveterinary value were collected from the wild. For example, out of 39 species, 29 species (74.36%) were collected from the wild (Table 3).

**Table 3:** Collection type of Baiku Yao veterinary plants.

**Collection type**	**Species no**.	**Species proportion (%)**
Wild	29	74.36
Wild or cultivated	3	7.69
Cultivated	7	17.95

Different plant parts are used in ethnoveterinary medicines, depending on the species. In the present study, whole plants and leaves (accounting for 28.21% each) were the most commonly used plant parts utilized in ethnoveterinary medicine preparations, followed by roots, branches, tubers, fruits, and leaves or fruits and branches (Table 4).

**Table 4:** Used parts of Baiku Yao veterinary plants.

**Used parts**	**Species no**.	**Proportion (%)**
Whole plants	11	28.21
Leaves	11	28.21
Roots	6	15.38
Branches and leaves	4	10.26
Tubers	3	7.69
Tuber-roots	2	5.13
Fruits and leaves	1	2.56
Branches, leaves and fruits	1	2.56
Total	39	100.00

### Veterinary Properties and Use Methods

**Figure 2:**
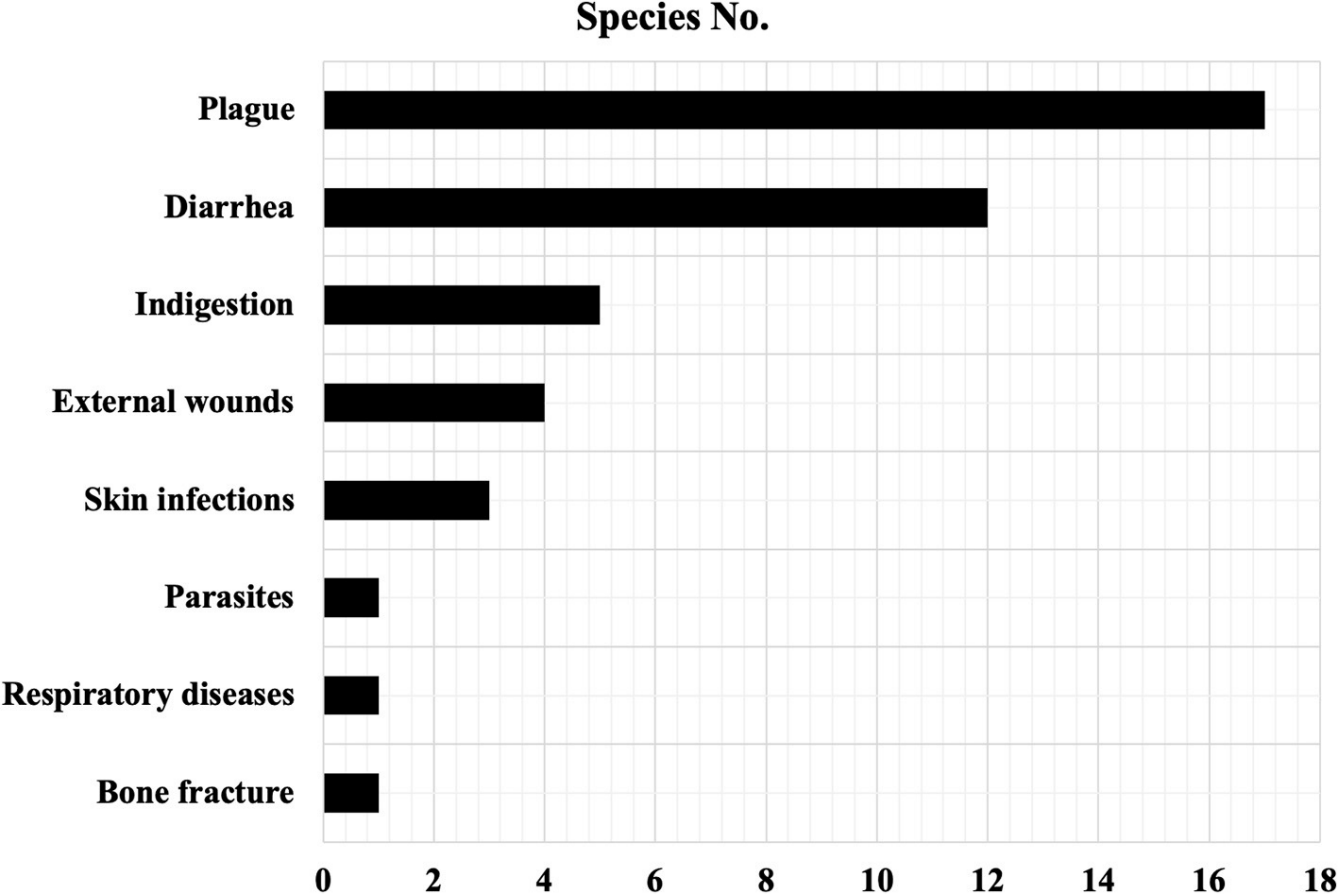
Disease types targeted by ethnoveterinary medicines in the Baiku Yao community.

Most of the ethnoveterinary plants we recorded were mainly used for treating cattle (74.4%) and pig (66.7%) diseases (Table 2). Raising pigs to supplement daily protein consumption and increase family income is very popular among the Baiku Yao community. However, cattle are raised for crop farming and local sacrifice activities. The identified ethnoveterinary plants are used mainly to treat eight animal diseases (Figure 2). Among them, the species used for the treatment of plague accounted for the highest proportion (43.6%), followed by diarrhea (30.8%), indigestion (12.8%), and external wounds (10.3%) (Figure 2). The results indicated that local people have deep veterinary knowledge, especially regarding the treatment of plague, diarrhea, and indigestion.

The animal plague mentioned in Table 2 is a highly contagious disease (such as African swine fever) that infects animals such as swine and cattle and can usually cause fever, anorexia, diarrhea, death, etc. Our interviewees were particularly impressed with the plants that prevent and treat animal plague because it had been only 2 years since the last large outbreak of African swine fever in mainland China, which led to significant losses among livestock keepers in neighboring communities. Therefore, the outbreak experience could be why local people always listed herbs as a priority treatment for animal plague. However, diarrhea and indigestion were the most common animal diseases locally, and almost no one used antibiotics for the treatment of these ailments. Therefore, the local people have knowledge about various plant species for the treatment of these two antibiotics.

The participants used plant decoctions or mashed plants that were then mixed with feed to treat diarrhea, indigestion and respiratory diseases. However, for skin infections, fractures and wounds, the plants are usually crushed and applied externally to infected or wounded areas or used as decoctions for livestock baths. According to the inhabitants, *Strobilanthes cusia* and *Gynura japonica* leaves, wine, salt, and mud mashed together are very effective for the external treatment of wounds. According to locals, the therapeutic effect of this formula is very promising, and it is worthy of further scientific verification and development.

Local inhabitants usually use ethnoveterinary plants both internally and externally to treat the animal plague. They spread the branches and leaves of *Paederia foetida, Tetradium ruticarpum*, and *Stachyurus chinensis* in pigpens or cowsheds and let the animals sleep on them to prevent or treat plague.

*Paederia scandens* and *T. ruticarpum* have very strong aromas and are reported to have positive biological activities, such as antimicrobial and anti-inflammatory activities (21, 22). Therefore, we speculate that the odder of these two species not only repels pests such as mosquitoes, preventing plague from spreading, but also provides some positive therapeutic effects. In addition, the leaves and small twigs of these species are also hung on the necks of dogs to treat plague. Although *S. chinensis* has no aroma, the leaves and small branches are used as “animal beds.” In traditional medicine, *S. chinensis* is used for clearing heat and urinary tract infections (23). However, to date, phytochemical studies on this species remain scarce; therefore, *S. chinensis*is could be worth investigating in the near future.

### The Evaluation of Veterinary Species

The study also used two indicators, the RFC and GR, to quantitatively evaluate the Baiku Yao's ethnoveterinary plant knowledge and the plants used for plague treatment. These two indicators help identify the most important plants to the local community, which have greater development potential.

In the current investigation, the highest RFC values were reported for *Curculigo capitulata* (RFC = 0.40), followed by *Stephania kwangsiensis* (0.38), *A. kwangsiensis* (0.32), *C. bungei* (0.32), *P. foetida* (0.26), and *T. ruticarpum* (0.24) (Table 2). According to interviews and our observations, these species share some similar characteristics, such as (i) large biomass and high abundance in the area; (ii) available in the wild but also in home gardens or wastelands near residents' houses; and (iii) good medicinal effects (higher ranking).

Based on the GR value, *S. cusia* (GR = 10) scored the highest, followed by *T. ruticarpum* (9) and *S. kwangsiensis* (9). *S. cusia*, and *T. ruticarpum* are reported to have antibacterial and antiviral properties (22, 24). Although *T. ruticarpum* has already been listed as an ethnoveterinary plant elsewhere (12), chemical or pharmacological studies on this species are lacking. Similarly, leaf extracts of *S. kwangsiensis* have been proven to kill bacteria and pests (25, 26), but no veterinary cases have been published for this species.

Plant species with higher GR values for plague treatment often have better RFCs, which further proves that these species play an important role in the treatment of livestock; therefore, further studies are required to validate this hypothesis, and various phytochemical investigations on these species are needed in the near future.

### Local Livestock Epidemic Prevention Regulations

**Figure 3:**
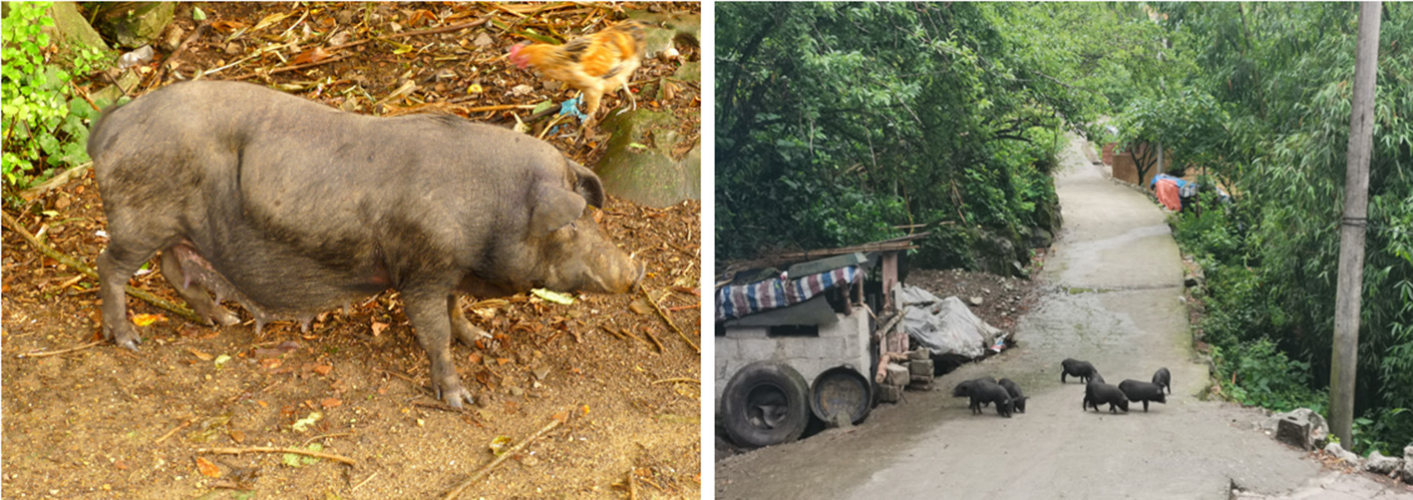
Black pigs kept by Baiku Yao people.

During the visit, we found that Baiku Yao villagers were very successful in preventing and controlling the African swine fever epidemic; thus, they were able to keep their animals healthy. Comparatively, there were almost no surviving domestic pigs from the swine fever outbreak in the surrounding Han and Zhuang villages at that time. These results confirm that the Baiku Yao people have unique and effective methods for the prevention and treatment of swine plague and cattle plague, which can be summarized into the following four points:

1) The Baiku Yao people generally prioritize indigenous breeds. For example, nearly 65% of the farmers we surveyed raised local black pigs (Figure 3). The local pigs are small, nimble, and have a mild fragrance, and their meat is considered delicious; thus, they are considered the best by the Baiku Yao people. Moreover, according to local people, these local pigs have higher disease resistance than common white pigs.
2) The Baiku Yao people still practice traditional breeding methods. Local people allow pigs to roam freely around the nearby areas (Figure 3), and they think that if the pigs have enough exercise, the meat will be more delicious, and the body will be healthier. In addition, most Baiku Yao people insisted on feeding wild vegetables or coarse grain to their pigs instead of market feeds. They believe that the market feeds have too many calories, leading to difficulty in digestion and the production of internal heat in pigs.
3) According to the participants, local professional pig farmers build pigpens in depressions far away from the village. They usually do not allow outsiders to enter the pig farms and sell pork to only community members, ensuring that pig farms are relatively independent. Moreover, physical isolation allowed the pig farms to avoid the impact of the African swine fever epidemic.
4) Baiku Yao inhabitants have deep knowledge about ethnoveterinary plants that can prevent and treat animal plague. Our results showed that more than 15 species were used to prevent and control animal plague. We even found that local people were using a mineral, Glauber's salt (Mirabilite), as a veterinary drug. According to one of our key informants, Dr. He, a local veterinarian, whenever he found pigs with indigestion and red skin, he added a certain amount of Glauber's salt to the pig food, allowing the pigs to recover quickly. Dr. He also mentioned that he used the same method during the African swine fever epidemic, and his piglets survived the epidemic. Glauber's salt is frequently used in traditional Chinese medicine and induces purging and laxative effects, clearing fire and reducing swelling (27).

The Baiku Yao people mainly use a primitive but effective way to avoid the occurrence of animal plague. In undeveloped parts of the world, there are still many livestock keepers raising animals on a small scale, such as family units. In some remote local communities, the use of modern animal drugs is not popular due to transportation issues and costs (28). Relying on the geographical location and surrounding environment, the traditional management model and natural herb usage can effectively reduce costs and promote effective results for animal rearing, which is typical in the Baiku Yao community.

## Suggestions

The excessive use of modern veterinary drugs usually shows promising results for a short period, but in the long term, they can cause various negative effects (29). For example, antibiotics can cause intestinal microbial disorders and ultimately reduce an animal's immunity (29). However, plant-based veterinary medicines usually do not have any side effects; therefore, they are considered safe. It is necessary to motivate people to use traditional veterinary medicine. Currently, there are only a few studies on traditional veterinary drugs, and they are mainly at the stage of cataloging and description. Therefore, it is important to conduct more research investment and provide additional funding to develop new safe and effective veterinary drugs based on traditional knowledge.

Disease prevention should always be the priority over disease treatment, and multiple measures can be applied, such as physical isolation or immunity boosting by using specific forage or natural medicines. Thus, there is an urgent need to develop and improve efficient community-based animal husbandry management to effectively address the frequent outbreaks of animal plague and zoonotic diseases. Baiku Yao village's management measures to prevent African swine fever could be used and replicated in other regions of China.

We also observed that some species serve more than one purpose. For example, most of the ethnoveterinary species we recorded can also be used as drugs to treat human diseases. These “multifunctional” economic potentials are worthy of further investigation. Moreover, some medicinal species, such as *Chirita tribracteata* var. *tribracteata* and *Polygonum nepalense* can also be used as regular fodder for livestock, according to Baiku Yao villagers. Promoting the domestication and cultivation of these multifunctional herbs and providing a market for local people to sell their ethnoveterinary products could not only improve the livelihood of local people but also serve as a step forward for sustainable utilization (30). Therefore, further studies in pharmacology, phytochemistry, and veterinary sciences based on ethnobotanical documents are required (31).

## Conclusions

The Baiku Yao people are dependent on medicinal plants for ethnoveterinary practices, and they have very deep ethnoveterinary knowledge. They have their own way of preventing and treating disease in their animals, which effectively reduces the effects of epidemics. Conserving and disseminating traditional ethnoveterinary knowledge is required to spread maintain this knowledge.

## Data Availability Statement

The original contributions presented in the study are included in the article, further inquiries can be directed to the corresponding author.

## Ethics Statement

The studies involving human participants were reviewed and approved by the Ethics Committee of Guangxi Institute of Traditional Medical and Pharmaceutical Sciences. Written informed consent for participation was not required for this study in accordance with the national legislation and the institutional requirements.

## Author Contributions

BL and RH conceived the study. RH, KL, and QH gathered the data. BL analyzed the data. BL drafted the first version of the manuscript and then reviewed and edited by AB, BL, and RH. All authors contributed to the article and approved the submitted version.

## Funding

This research was funded by the National Natural Science Foundation of China (32000264), the Natural Science Foundation of Guangxi, Grant Number (2018GXNSFBA281162), the Open Program of Guangxi Key Laboratory of Traditional Chinese Medicine Quality Standards (202007), Project of Ecological Cultivation and Quality Assurance of Authentic Medicine in Guangxi Province subsidized by the Central Government in 2021 (Guicaishehan(2021)138), Guangxi Chinese Medicine Key Disciplines Construction Projects (GZXK-Z-20-69), and the Special Project of Lushan Botanical Garden of Chinese Academy of Sciences (2021ZWZX12).

## Conflict of Interest

The authors declare that the research was conducted in the absence of any commercial or financial relationships that could be construed as a potential conflict of interest.

## Publisher's Note

All claims expressed in this article are solely those of the authors and do not necessarily represent those of their affiliated organizations, or those of the publisher, the editors and the reviewers. Any product that may be evaluated in this article, or claim that may be made by its manufacturer, is not guaranteed or endorsed by the publisher.

## References

[1] He P Baiocchi G Hubacek K Feng K Yu Y. The environmental impacts of rapidly changing diets and their nutritional quality in China. Nat Sustain. (2018) 1:122–7. 10.1038/s41893-018-0035-y30999146

[2] Morgan N Prakash A. International livestock markets and the impact of animal disease. Rev Sci Tech. (2006) 25:517–28. 10.20506/rst.25.2.1685 17094694

[3] DiAz-Cruz MS De Alda MaJL Barcelo D. Environmental behavior and analysis of veterinary and human drugs in soils, sediments and sludge. TrAc-trend Anal Chem. (2003) 22:340–51. 10.1016/S0165-9936(03)00603-4

[4] Boxall AB. The environmental side effects of medication: how are human and veterinary medicines in soils and water bodies affecting human and environmental health? EMBO Rep. (2004) 5:1110–6. 10.1038/sj.embor.7400307 15577922 PMC1299201

[5] Reig M Toldrá F. Veterinary drug residues in meat: concerns and rapid methods for detection. Meat Sci. (2008) 78:60–7. 10.1016/j.meatsci.2007.07.029 22062096

[6] Shao Y Wang Y Yuan Y Xie Y. A systematic review on antibiotics misuse in livestock and aquaculture and regulation implications in China. Sci Total Environ. (2021) 2021:149205. 10.1016/j.scitotenv.2021.149205 34375247

[7] Woolhouse M Ward M Van Bunnik B Farrar J. Antimicrobial resistance in humans, livestock and the wider environment. Philos T R Soc B. (2015) 370:20140083. 10.1098/rstb.2014.0083 25918441 PMC4424433

[8] Chomel BB Kasten R. Bartonellosis, an increasingly recognized zoonosis. J App Microbiol. (2010) 109:743–50. 10.1111/j.1365-2672.2010.04679.x 20148999

[9] Hu YJ Cowling BJ. Reducing antibiotic use in livestock, China. B World Health Organ. (2020) 98:360. 10.2471/BLT.19.243501 32514201 PMC7265937

[10] Abo-El-Sooud K. Ethnoveterinary perspectives and promising future. Int J Vet Sci Med. (2018) 6:1–7. 10.1016/j.ijvsm.2018.04.001 30255071 PMC6145062

[11] Wanzala W Zessin K Kyule N Baumann M Mathia E Hassanali A. Ethnoveterinary medicine: a critical review of its evolution, perception, understanding and the way forward. Livest Res Rural Dev. (2005) 119:17.

[12] Xiong Y Long C. An ethnoveterinary study on medicinal plants used by the Buyi people in Southwest Guizhou, China. J Ethnobiol Ethnomed. (2020) 16:1–20. 10.1186/s13002-020-00396-y 32807192 PMC7433110

[13] Liu J Liu B Shan B Wei S An T Shen G. Prevalence of African swine fever in China, 2018-2019. J Med Virol. (2020) 92:1023–34. 10.1002/jmv.25638 31769521

[14] Luo Z Yang J Yu Y Rong J Cen L Ren G. Study on physical anthropology of the Baiku Yao population in China. Acta Anthropol Sin. (2003) 12:2155–6. Available online at: http://www.anthropol.ac.cn/EN/Y2003/V22/I02/150

[15] Qiu F. Cultural changes and recognition: a survey of the current situation of Baiku Yao culture in Nandan County, Guangxi Province. Econ Sco Dev. (2008) 6:110–5. Available online at: http://www.cqvip.com/qk/87674x/200802/1000040990.html

[16] Martin GJ. Ethnobotany: A Methods Manual. London: Routledge (2010).

[17] Ahmad M Sultana S Fazl-I-Hadi S Ben Hadda T Rashid S Zafar M. An ethnobotanical study of medicinal plants in high mountainous region of Chail Valley (District Swat-Pakistan). J Ethnobiol Ethnomed. (2014) 10:1–18. 10.1186/1746-4269-10-36 24739524 PMC4022037

[18] Russo EB. Headache treatments by native peoples of the Ecuadorian Amazon: a preliminary cross-disciplinary assessment. J ethnopharmacol. (1992) 36:193–206. 10.1016/0378-8741(92)90044-R 1434677

[19] Huai H Pei S. Common methods in ethnic medicine research. Chin J Ethnomed Ethnopharm. (2000) 43:63–6. Available online at: http://www.cqvip.com/qk/97965x/200002/4323292.html

[20] Bessada SM Barreira JC Oliveira MBP. Asteraceae species with most prominent bioactivity and their potential applications: a review. Ind Crop Prod. (2015) 76:604–15. 10.1016/j.indcrop.2015.07.073

[21] Wang L Jiang Y Han T Zheng C Qin L. A phytochemical, pharmacological and clinical profile of Paederia foetida and *P*. scandens. Nat Prod Commun. (2014) 9:1934578X1400900640. 10.1177/1934578X1400900640 25115105

[22] Li M Wang C. Traditional uses, phytochemistry, pharmacology, pharmacokinetics and toxicology of the fruit of *Tetradium ruticarpum*: a review. J Ethnopharmacol. (2020) 263:113231. 10.1016/j.jep.2020.113231 32758577

[23] Sun J Li C Ma Z Wang X Zhang Y. Chemical constituents of *Stachyurus chinensis*. Chem Nat Comp. (2021) 57:390–1. 10.1007/s10600-021-03368-0

[24] Yu H Li T Ran Q Huang Q Wang J. *Strobilanthes cusia* (Nees) Kuntze, a multifunctional traditional Chinese medicinal plant, and its herbal medicines: a comprehensive review. J Ethnopharmacol. (2021) 265:113325. 10.1016/j.jep.2020.113325 32889034

[25] Deng Y Xu H. Insecticidal activities and active ingredients of *Stephania kwangsiensis* L. Sci Agr Sin. (2005) 38:523–7. Available online at: http://delphacid.s3.amazonaws.com/7195.pdf

[26] Deng Y Li J Gao C Yang L. Inhibitory activity of the extract from the root tubers of *Stephania kwangsiensis* and its compounds against pathogenic fungi and bacteria. Plant Protect. (2006) 32:43–6. Available online at: http://www.plantprotection.ac.cn/ch/reader/create_pdf.aspx?file_no=20060411&flag=1&journal_id=zwbh&year_id=2006

[27] Ying B Zhang W Zhang Z. Pharmacological action study of mirabilite. Mod J Integr Tradit Chin West Med. (2003) 12:2155–6. Available online at: http://xdjh.chinajournal.net.cn/WKC/WebPublication/paperDigest.aspx?paperID=0e29c427-565e-490b-b9bf-a8034df93973#

[28] Suroowan S Javeed F Ahmad M Zafar M Noor MJ Kayani S. Ethnoveterinary health management practices using medicinal plants in South Asia – a review. Vet Res Commun. (2017) 41:147–68. 10.1007/s11259-017-9683-z 28405866

[29] Clement M Olabisi M David E Issa M. (2019). Veterinary pharmaceuticals and antimicrobial resistance in developing countries. In Bekoe S.O Saravanan M editors. Veterinary Medicine and Pharmaceuticals. London: IntechOpen Limitd. (2019). 10.5772/intechopen.84888 Available online at: https://www.intechopen.com/chapters/66512

[30] Ahmad S Zafar M Shinwari S Ahmad M Shinwari ZK Sultana S. Ethno-medicinal plants and traditional knowledge linked to primary health care among the indigenous communities living in western hilly slopes of Dera Ghazi Khan, Pakistan. Pak J. Bot. (2020) 52:519–30. 10.30848/PJB2020-2(19)

[31] Ahmad M Malik K Tariq A Zhang G Yaseen G Rashid N. Botany, ethnomedicines, phytochemistry and pharmacology of Himalayan Paeony (*Paeonia emodi* Royle.). J Ethnopharm. (2018) 220:197–219. 10.1016/j.jep.2018.04.004 29625273

